# Supplementary material for: Epigenetic Modulation of GPER Expression in Gastric and Colonic Smooth Muscle of Male and Female Non-Obese Diabetic (NOD) Mice: Insights into H3K4me3 and H3K27ac Modifications
Source: Int J Mol Sci. 2024 May 11;25(10):5260. doi: 10.3390/ijms25105260 (PMC11121689; doi:10.3390/ijms25105260)
Supplement: Supplementary file 1 [file ijms-25-05260-s001.zip › ijms-2917089-supplementary.pdf]

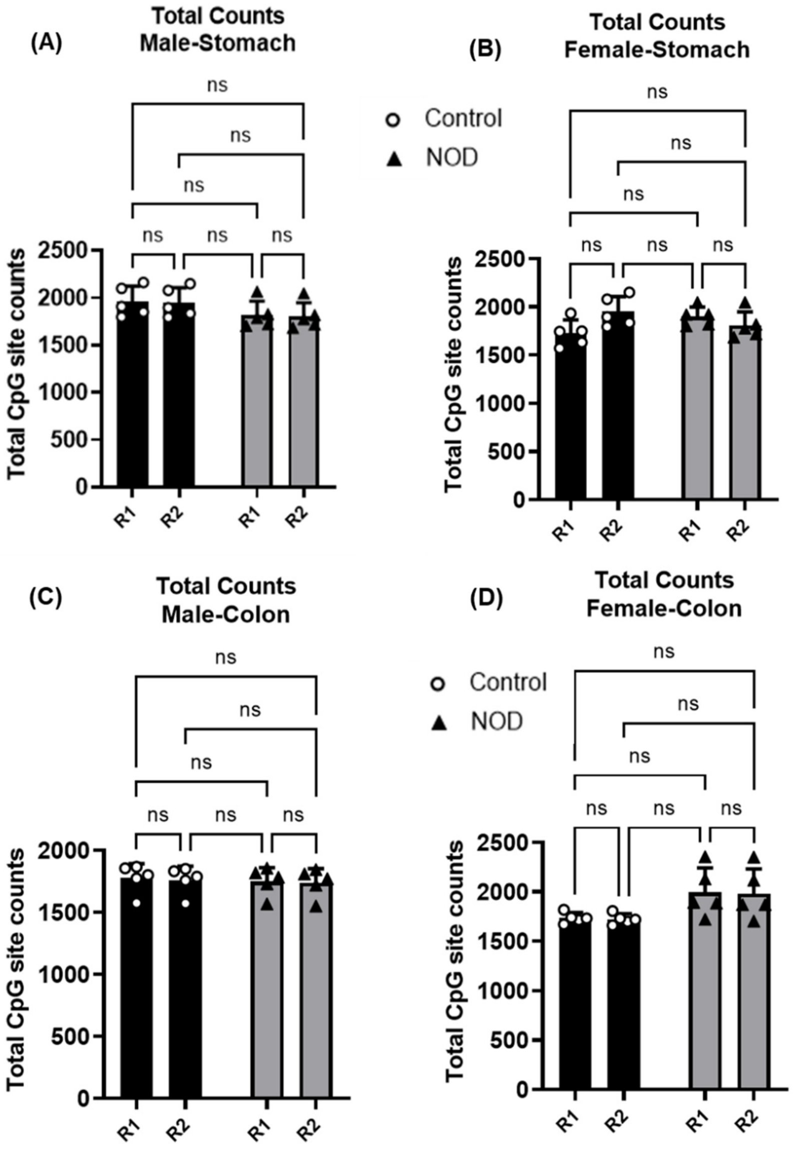

Supplementary Figure S1: Comparison of total CpG site counts at region R1 vs R2 in mouse CTR versus NOD male and female gastric and colonic smooth muscle tissues. *A and B:* CpG site counts between R1 and R2 in control gastric smooth muscle compared to NOD gastric smooth muscle were similar in both male and female mice. *C and D:* CpG site counts between R1 and R2 in control colonic smooth muscle compared to NOD colonic smooth muscle were similar in both male and female mice. Results were deemed significant when p <0.05. 2-way ANOVA analysis p-value (nsp>0.05) n=5

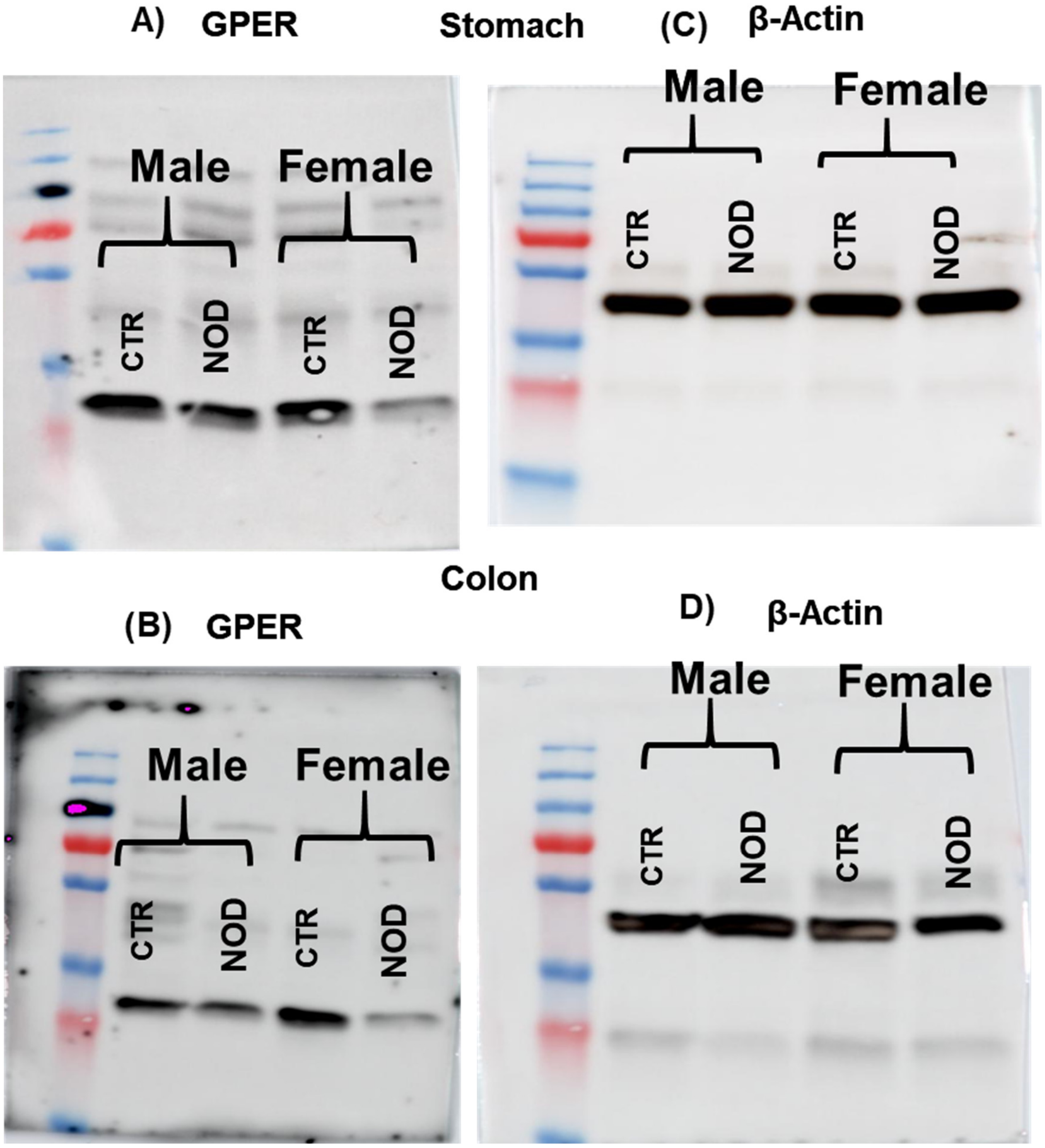

Supplementary Figure S2: GPER protein expression in the gastric and colonic smooth muscles: Western blot analysis showing the protein content of GPER (A and B) compared to β-actin (C and D) in the gastric (top) and colonic (bottom) smooth muscles of the CTR versus NOD male and female mice in 1 cohort of 4 biologically-independent experiments. These are the full blots of Figure 5 inserts.

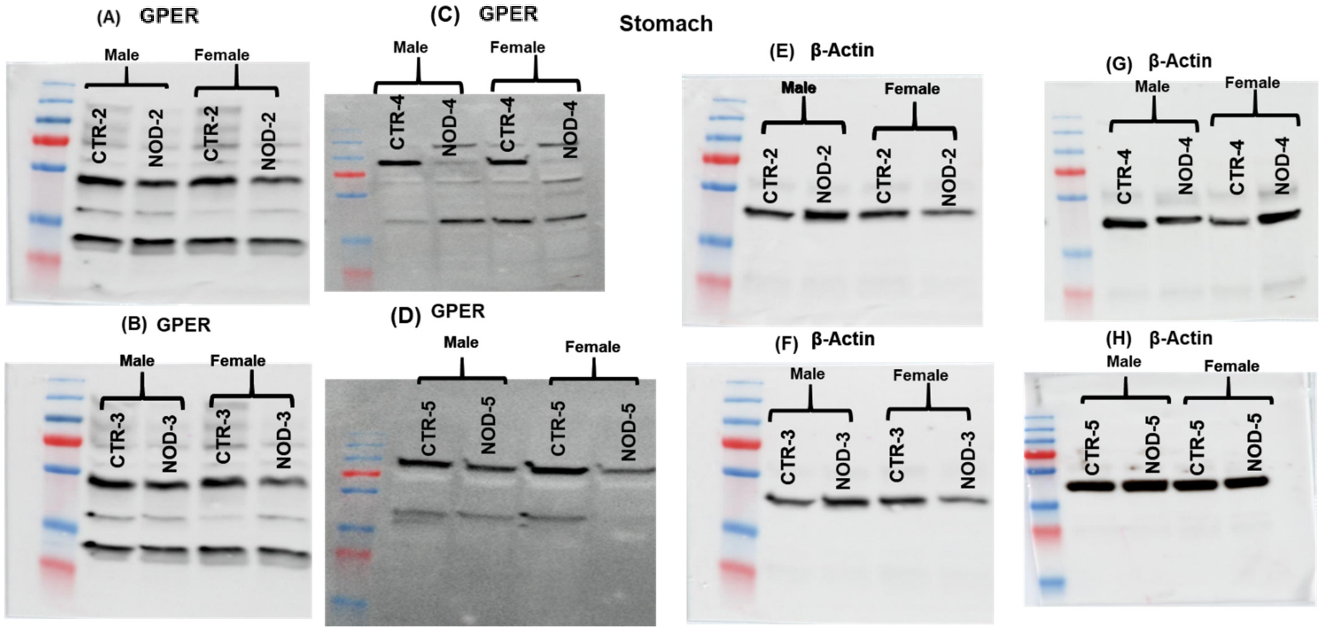

Supplementary Figure S3: GPER protein expression in the gastric smooth muscles: Western blot analysis showing the protein content of GPER (A-D) compared to β-actin (E-H) in the gastric smooth muscles of the CTR versus NOD male and female mice in 4 cohorts of 4 biologically-independent experiments equaling total of 16 animals.

Colon

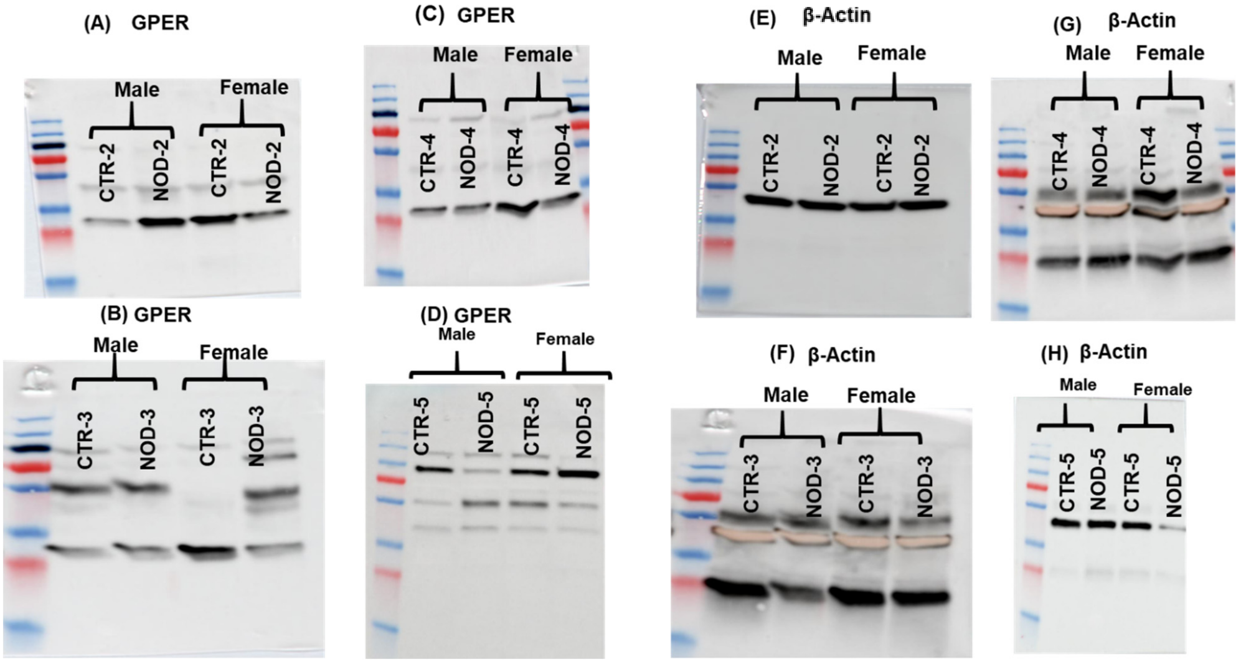

Supplementary Figure S4: GPER protein expression in the colonic smooth muscles: Western blot analysis showing the protein content of GPER (A-D) compared to β-actin (E-H) in the colonic smooth muscles of the CTR versus NOD male and female mice in 4 cohorts of 4 biologically-independent experiments equaling total of 16 animals.
